# Effect of application of fibrin glue on vocal fold healing after surgical manipulation in rabbits

**DOI:** 10.1016/j.bjorl.2021.04.005

**Published:** 2021-05-06

**Authors:** Fernanda da Silva Santos, Felipe Hideo Ikeda, Mirna Duarte Barros, André de Campos Duprat

**Affiliations:** a Irmandade da Santa Casa de Misericórdia de São Paulo, Departamento de Otorrinolaringologia, São Paulo, SP, Brazil; b Faculdade de Ciências Médicas da Santa Casa de São Paulo, Departamento de Morfologia, São Paulo, SP, Brazil

**Keywords:** Larynx, Fibrin tissue adhesive, Rabbits, Vocal cords, Fibrosis

## Abstract

**Introduction:**

A challenge in phonosurgery is achieving good vocal quality with minimal vocal fold fibrosis. Fibrin glue can be applied to minimize fibrosis; however, its use in the larynx is based primarily on clinical experience, particularly in extensive lesions.

**Objective:**

The objective of this study was to evaluate the effect of application of fibrin glue on collagen concentration at the late phase of the healing process after detaching a pedicled flap of the vocal fold cover in rabbits.

**Methods:**

In this prospective animal study, twelve adult male rabbits underwent laryngeal microsurgery, in which an incision was made along the entire length of both vocal folds, followed by extensive mucosal detachment and section of the incision ends. Fibrin glue was applied in the left vocal fold, and the mucosa was repositioned. In the right vocal fold, the mucosa was repositioned without treatment with fibrin glue. After 3-months, the rabbits were euthanized. Histological analyses were performed, and the data collected were subjected to statistical analysis.

**Results:**

Vocal folds treated with fibrin glue presented higher collagen concentration in Masson trichrome staining and significantly higher (*p* < 0.05) collagen concentration in picrosirius red staining compared to control vocal folds.

**Conclusion:**

Treatment with fibrin glue led to greater vocal fold fibrogenesis in the present study. Nonetheless, further studies are necessary to determine the prophylactic effect of sealants in laryngeal surgeries that require extensive detachment of the vocal fold cover.

## Highlights

• Evaluation of prophylactic effect of sealants in laryngeal surgeries that require extensive detachment of the vocal fold cover Prophylactic effect of sealants in laryngeal surgeries.
• Incision over the entire length of the vocal fold, followed by anterior and posterior sectioning of the mucosa, creating a pedicled flap. Extensive detachment of the vocal fold cover creating a pedicled flap.
• Fibrin glue induced greater fibrogenesis in the vocal fold in a later stage of the healing process. Fibrin glue induced greater fibrogenesis in the vocal fold.

## Introduction

The vocal fold (VF) is composed of stratified squamous epithelium, lamina propria (LP), and muscle tissue. Hirano introduced the concept of the “cover,” formed by the epithelium and superficial layer of the LP, which slides over the “body” (intermediate and deep layers of the LP, also known as the vocal ligament, and vocal muscle), allowing the VFs to vibrate.^1^, ^2^ The presence of fibrosis on the cover could interfere with vibration, causing dysphonia.

A challenge in phonosurgery is achieving good vocal quality with minimal VF fibrosis. Current treatments for VF scarring often yield unsatisfactory outcomes.^3^ Therefore, it is essential to investigate mechanisms to prevent VF fibrosis.

The best surgical results are obtained when there is better preservation of a healthy structure of the vocal fold LP, decreasing the inflammatory response and scar tissue formation.^4^, ^5^ Microflap is a well-established technique for removing benign lesions,^4^ which consists of detaching the superficial layer of the LP without damaging the vocal ligament, minimizing scar tissue formation. In some cases, the lesions may affect a large portion of the VF, possibly increasing fibrosis due to more significant surgical manipulation.

The use of fibrin glue (FG) could minimize surgery-generated fibrosis. It can be applied in the VF for attaching mucosal flaps and placing grafts in Reinke’s space to improve healing.^6^, ^7^ Nonetheless, few studies have evaluated its effectiveness, and, in clinical practice, FG is used according to surgeon’s experience.

Portes et al. reported FG’s effect on VF healing of pigs after placing the sealant in a pocket created in the VF and observed that the area occupied by collagen fibers was higher in VFs that were fixed with the sealant than in VF pockets without sealant.^7^ Scapini et al. compared VF healing in rabbits after placing a fascia graft in the presence and absence of FG and observed that collagen fiber concentration increased around the glued fascia 90-days post-placement.^8^

These studies evaluated the FG effect on flaps created in small areas of the VF. Nonetheless, no studies have analyzed the healing process in extensive lesions, which affects the epithelium of the full length of the VFs. In extensive lesions, there is a more significant loss of normal VF mucosa during surgery, leaving areas of vocal ligament exposed, which can result in more scarring. In these situations, it is necessary to cover the exposed vocal ligament with the remaining epithelium in order to decrease fibrogenesis. Sealants help join the incision margins, potentially decreasing fibrogenesis, compared to joining the epithelium without any assistance.

The present study evaluated the effect of FG on extensive VF procedures in a later phase of the healing process.

## Methods

The Animal Research Ethics Committee approved this study (Protocol nº 2151-14). All animal care procedures were performed according to Brazilian Federal Law (11974/2008) and the guidelines of the Brazilian College on Animal Experimentation.

Twelve healthy adult male New Zealand rabbits weighing 3.5–4.5 kg were selected. Animals that presented signs and symptoms of preexisting conditions, developed complications during the observation period or died during that period were excluded. Each animal received atropine 0.1 mg/kg subcutaneously, acepromazine 0.1 mg/kg, ketamine 40 mg/kg and xylazine 10 mg/kg intramuscularly as pre-anesthetic medication and were anesthesized with ketamine 20 mg/kg and diazepam 4 mg/kg. Each rabbit was positioned supine with cervical hyperextension. Cervical trichotomy and asepsis of the cervical region were performed immediately before surgery.

**Figure 1:**
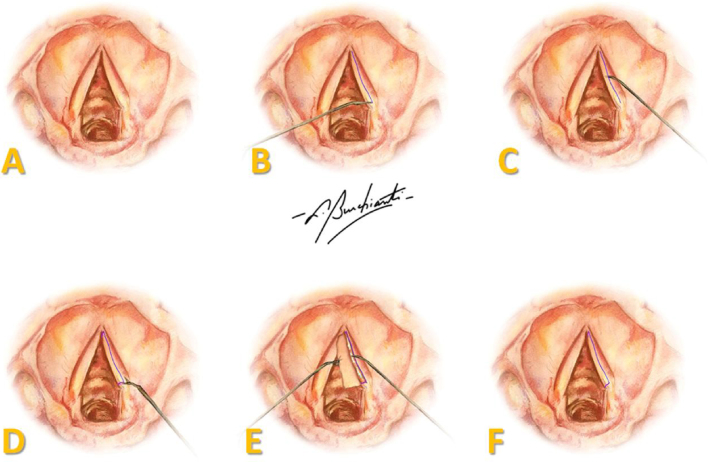
Representation of the surgical procedure in human vocal folds: (A) endoscopic view of VFs; (B) incision in the superior aspect of the full length of the VF; (C) mucosal detachment; (D) sectioning of the mucosa; (E) pedicled flap with FG on the vocal ligament; (F) repositioning of the flap over the vocal ligament. Art by Burchianti LC.

A two centimeter anterior cervical midline incision was made, and tissue was retracted to the perichondrium of the thyroid cartilage. A laryngofissure was done from the middle of the thyroid cartilage until the cricothyroid membrane and, under microscopy, an incision was made in the superior aspect over the entire length of both VFs, followed by extensive mucosal detachment and anterior and posterior sectioning of the mucosa, creating a pedicled flap (Fig. 1).

In the left VF, 0.1 mL of Evicel® (Ethicon Inc., Somerville, NJ) FG (fibrinogen, 55–85 mg/mL; thrombin, 800–1200 UI/mL; and calcium chloride, 5.6–6.2 mg/mL) was applied followed by flap repositioning. In the right VF, the flap was positioned without FG. The cartilage, cervical musculature, and skin were repaired using 5.0 Prolene suture.

After the procedure, the animals were observed for a three-month period followed by euthanasia. The surgical specimen (structures from epiglottis until the first tracheal ring) was removed through the site of the incision and fixed in 10% formaldehyde solution.

The larynx was isolated and sectioned into right and left hemilarynx. After identifying the VFs macroscopically, the tissue comprising 3-millimeters (mm) of the supraglottis, VF, and 3-mm of the subglottis was removed, sent to the Department of Morphology for processing. Tissue sections from each VF were prepared and stained with hematoxylin and eosin (HE), to determine the precise location of the VFs and the phase of the inflammatory process, and picrosirius red and Masson’s trichrome, to determine the area occupied by collagen in the LP of each VF.

The Masson’s trichrome and picrosirius red stained laminas were scanned and the images were saved in Pannoramic viewer software (3D HISTECH Ltd.) at 5× magnification. Color parameters and units of measurement were adjusted in Image Pro-Plus 4.5 software (Media Cybernetics), and the areas to be analyzed, which consisted of the subepithelial region (without the vocal muscle), were manually delimited. The software calculated the percentage of area occupied by collagen fibers in the LP of the VFs.

Quantitative characteristics were described by mean, standard deviation, median, minimum, and maximum and were compared between left and right side using a paired Wilcoxon signed-rank test^9^; *p*-values less than 0.05 were considered significant.

## Results

Six animals were lost during the 3-month follow-up period: one rabbit at 9-days post-operative due to dehydration, and five due to cervical abscesses at the incision site: four at 60-days and one at 90-days post-operative. Thus, only 6 rabbits were included in the current study.

**Figure 2:**
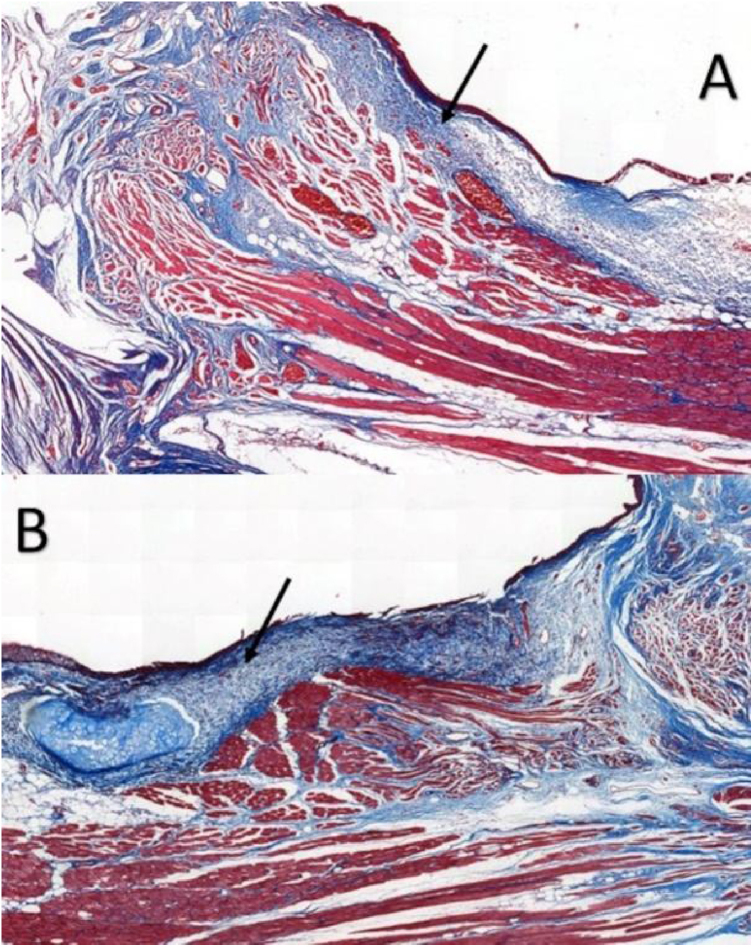
Masson trichrome-stained sections in rabbits at a magnification of 5×: (A) control VF; (B) VF with fibrin glue. Arrows point to the subepithelial region of the VFs. Note higher concentration of collagen in part B, in blue.

Six larynges were evaluated. Masson’s trichrome-stained tissue sections determined the area occupied by collagen (Fig. 2).

In VFs from the intervention group, the values ranged from 11.593% to 81.552%, with an average of 26.619%, whereas VFs from the control group ranged between 3.700%–34.026%, with an average of 17.783%; the differences were not significant (*p* = 0.917) (Table 1).

**Table 1:** Area of collagen fibers in Masson’s trichrome-stained sections. Data analyzed using the paired Wilcoxon rank-signed test.

	Fibrin glue fixed vocal fold (n = 6)	Control vocal fold (n = 6)	*p*-Value
Masson trichrome			0.917
(%) mean ± SD	26.6 ± 27	17.8 ± 11	
(%) median (min; max)	17 (11.6; 81.6)	18.5 (3.7; 34.0)	

**Figure 3:**
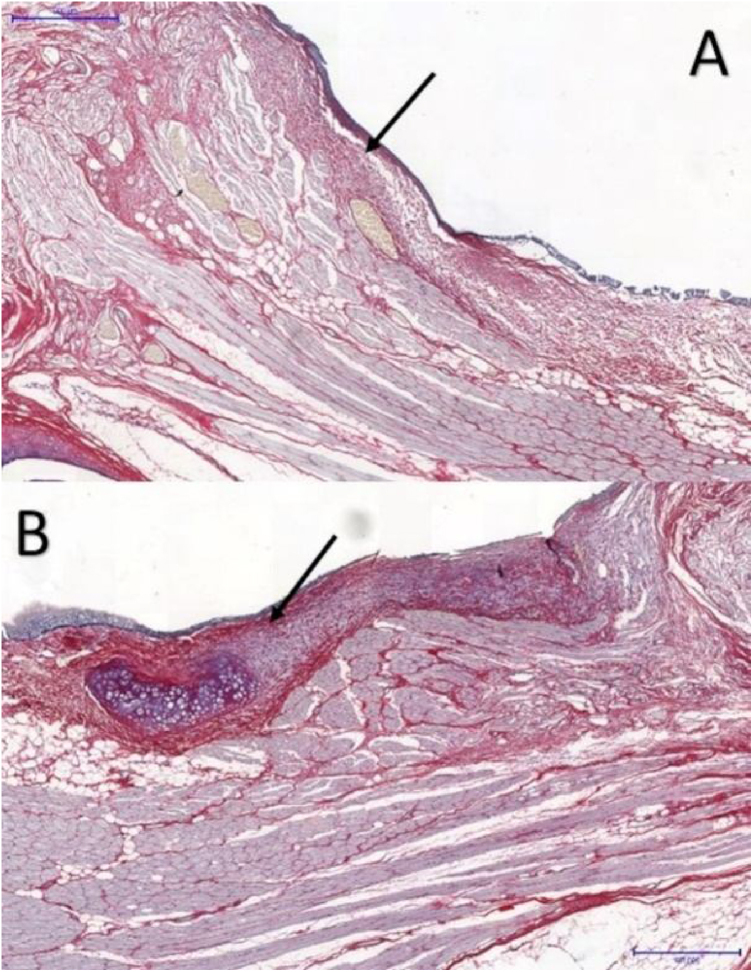
Picrosirius red-stained tissue sections at a magnification of 5×: (A) control VF; (B) VF with FG. Arrows point to the subepithelial region of the VFs. Note higher concentration of collagen in part B, in red.

The area occupied by collagen fibers was also determined in picrosirius red-stained sections (Fig. 3).

In VFs with FG, the area occupied by collagen fibers ranged from 28.261% to 58.7935%, with an average of 37.494%, whereas in the control group, the percentage ranged from 12.420% to 44.933%, with an average of 20.259%. These differences were significant (*p* = 0.046) (Table 2).

**Table 2:** Area occupied by collagen fibers in picrosirius-stained tissue sections. Data analyzed using the paired Wilcoxon rank-signed test.

	Fibrin glue fixed vocal fold (n = 6)	Control vocal fold (n = 6)	*p*-Value
**Picrosirius**			0.046
(%) mean ± SD	37.5 ± 11.9	20.3 ± 12.5	
(%) median (min; max)	32.8 (28.3; 58.8)	15.7 (12.4; 44.9)	

## Discussion

In contrast with other studies,^4^, ^7^, ^8^, ^10^^,^^11^ the current study evaluated the long-term effect of FG in scar tissue formation (collagen) following extensive detachment of the VF cover (microflap). It has been postulated that FG could minimize scarring of the vocal folds in procedures such as extensive Reinke’s edema removal and major stria sulcus surgeries, but so far, the animal model studies have not supported such findings.^7^, ^8^, ^11^

Rabbits were chosen because of their availability, larynx size, low vocalization, and low cost.^12^, ^13^ Additionally, rabbits’ VFs are distributed in three layers with a gradual increase in collagen fiber concentration,^12^ as observed in humans. Male rabbits were selected because they have a larger larynx and allow avoidance of hormonal variations during the healing process.

The procedure was performed via laryngofissure because this technique gives a better visualization of laryngeal structures, allows more precise manipulation of tissues, and is a well-established approach to access the larynx in rabbits.^14^ However, laryngofissure is more invasive than endoscopic techniques and may cause more complications.^15^ In the present study, laryngofissure was performed on twelve rabbits, and there were six deaths, five of them due to cervical abscess over 60-days after the procedure. These complications were mainly attributed to skin infections transmitted between rabbits that were confined together during the study period. When symptoms of infection first appeared, the sick animals were isolated, but the transmission could have happened in a subclinical phase.

For this study, rabbits were euthanized three months after surgery to evaluate mature scars and the long-term effects of treatment with FG. Although Rousseau et al. indicated that studies should evaluate long-term healing of vocal folds in rabbits for 6-months,^10^ this significantly increases costs and the possibility for complications. Moreover, the increase in collagen fibers and its organization in large bundles observed by Rousseau et al. may occur with an evaluation time ranging from three to 6 months. Portes et al. examined the VFs of six pigs three months after surgery,^7^ and Scapini et al. evaluated the effects of fascia placement in the presence and absence of FG within 90-days of the procedure.^8^ Moreover, Maunsell et al. assessed the short-term and long-term effects of treatment with FG and suturing on VF healing after 7 and 90-days, respectively.^11^

Previous studies evaluated VF healing after applying the microflap technique in a small area of the VF, creating a pocket to apply the sealant and, posteriorly, assessing its prophylactic effect on scarring.^4^, ^7^ In this study, the injury created affected the entire anteroposterior region of the VF, followed by extensive mucosal detachment and section of the anterior and posterior edges of the incision. In clinical practice, this situation may occur during surgery of the sulcus vocalis, large cysts or excision of Reinke’s edema and is more likely to generate large scars, which compromises VF vibration, thus, producing poor vocal results.

In cases requiring greater surgical manipulation of the VF, the VF layers should be placed close to the natural position, without dead spaces, to allow healing. Several scarring minimization options have been evaluated for better vocal outcomes. FG is widely used clinically in other specialties and has been applied in laryngeal surgeries with major anatomical disorders. Nonetheless, there is little scientific evidence to justify its extensive use and high cost. A recent systematic review evaluated the effect of FG on the healing of gastrointestinal anastomoses, and seven studies found positive effects, whereas eight studies found adverse effects.^16^ The reported benefits included improvement of the mechanical properties of the tissue, but not improvement in healing.^16^ The former was expected in the present study because FG provides better tissue accommodation between the vocal fold layers; however, these results were not corroborated by histological findings.

H and E staining was used to allow the identification of tissue, showing the precise location of the epithelium of the VF; identification of cellular architecture and characteristics of the inflammatory infiltrate, indicating a remodeling-stage inflammatory process. Once those structures were identified, we were able to apply the other staining methods for collagen quantification, the scope of the present study.

Masson’s trichrome is widely used for visualizing tissue fibrosis.^17^ Histological analysis indicated that collagen areas were not significantly more abundant in VFs with FG, which can be attributed to limitations in the quantitative analysis by Masson’s trichrome staining, since this staining poses greater difficulty for the image processing software’s available in distinguishing between the red-stained healthy tissue and blue-stained fibrotic tissue.^17^

Picrosirius red has high sensitivity and specificity for collagen detection^18^; it is widely used in VF healing studies,^7^, ^8^, ^10^ allowing collagen distribution and organization to be determined,^18^ and helping to differentiate between the types of collagen present in different layers by immunohistochemistry.

In this study, the VFs with FG showed significantly larger areas occupied by collagen fibers (*p* < 0.05), in accordance with the results of Portes et al., wherein vocal fold fibroplasia was significantly greater in tissues with FG.^7^ Maunsell et al. observed that collagen concentration was higher in VFs with FG without statistical significance.^11^ Furthermore, Scapini et al. observed a significant increase in collagen concentration around the fascia in VFs with FG 90-days after the procedure,^8^ which is consistent with the present study.

Scapini et al. attributed this result to the higher expression of Transforming Growth Factor β1 (TGF-β1) in the presence of FG.^8^ TGF-β1 is a cytokine that regulates healing and plays an essential role in activating fibroblasts, which are abundant in vocal scars.^19^ Saed et al. evaluated potential changes in TGF-β1 expression by applying FG in mesothelial cells of the human peritoneum and observed that expression decreased, whereas the concentration of types I and III collagen increased, potentially reducing the chance of adhesions in abdominal surgeries.^20^

Saed et al. evaluated the effect of FG Tisseel® (Baxter Healthcare Corp., Westlake Village, CA) on TGF-β1 expression at different combination of components and observed that the preparation without aprotinin showed a higher reduction in TGF-β1 expression, which could reduce fibrogenesis.^20^ Evicel® (Ethicon Inc., Somerville, NJ) does not contain aprotinin and, therefore, could have a reduced TGF-β1 expression; however, Saed et al. used peritoneal tissue, which does not have the same characteristics as the vocal fold. Other studies used Tissucol® (Baxter, Immuno AG, Vienna, Austria),^7^, ^8^, ^11^ which has the same composition as Tisseel® (Baxter Healthcare Corp., Westlake Village, CA), with similar results to the present study, suggesting that the difference in glue composition was not a significant contributor to VF healing.

## Conclusion

VFs with FG after detachment of pedicled graft showed more extensive area occupied by collagen fibers than the control VFs in rabbits. Therefore, in the present study, the glue induced greater fibrogenesis in the vocal fold in a later phase of the healing process.

## Funding

The 10.13039/501100002322Coordination for the Improvement of Higher Education Personnel (CAPES) and the 10.13039/501100001807São Paulo Research Foundation (FAPESP) funded this research (Grant #2014/10767-0) (Process nº 2014/10767-0).

## Conflicts of interest

The authors declare no conflicts of interest.

This study was performed in accordance with the Brazilian Federal Law (11974/2008) and the guidelines approved by the Brazilian College of Animal Experimentation (COBEA).
